# A Gratifying Appointment

**Published:** 1869-07-15

**Authors:** 


### *A Gratifying Appointment.*

Readers of the JOURNAL will recollect that a year or two since we spoke highly of the efforts of Rev. E. O. Haven,

D.D., LL.D., President of the University of Michigan, for his powerful and unanswerable argument against the introduction of a homœopathic chair into the medical department of that Institution. The Profession of the Northwest owe him a debt of gratitude for warding off, for the time being at least, so foul a disgrace from a medical college. We are gratified, exceedingly, in noticing that he has been elected President of the Northwestern University, at Evanston, our beautiful neighboring suburban town. He has resigned his position at Ann Arbor, and will soon enter upon the discharge of the duties of his new position. Although not nominally, yet in fact, Evanston constitutes a part of Chicago, and we may, therefore, claim him as one of our own citizens. He brings to his new place large practical experience as a teacher, great executive abilities, a mind profound and logical, yet brilliant and versatile. An accomplished scholar, writer and orator, he combines elegant culture and social refinement with an unassuming amiability of disposition, and genial manners, which can not fail to make him universally popular, as well as successful.

We congratulate the University on securing his services, and cordially welcome him to his enlarged and most important sphere of usefulness.

### *Locations.*

In common, we suppose, with our editorial *confreres*, we are in the receipt of numerous letters requesting our advice as to "openings for practice," either in this city or in some growing and enterprising western town. In most instances a partnership is desired with some "well-established practitioner."

Briefly—we know of no such chances. The only way to get at an "opening" is to make it. Find a place that suits, and, if merited, success will, sooner or later, follow. "Cold comfort"—but the best in our possession.

